# Immune-mediated cardiac development and regeneration

**DOI:** 10.1016/j.semcdb.2025.103613

**Published:** 2025-05-01

**Authors:** Timothy C. Byatt, Ehsan Razaghi, Selin Tüzüner, Filipa C. Simões

**Affiliations:** Institute of Developmental and Regenerative Medicine, Department of Physiology, Anatomy and Genetics, University of Oxford, Oxford, United Kingdom

**Keywords:** Heart, Immune Cells, Development, Regeneration, Cardiac Niche

## Abstract

The complex interplay between the immune and cardiovascular systems during development, homeostasis and regeneration represents a rapidly evolving field in cardiac biology. Single cell technologies, spatial mapping and computational analysis have revolutionised our understanding of the diversity and functional specialisation of immune cells within the heart. From the earliest stages of cardiogenesis, where primitive macrophages guide heart tube formation, to the complex choreography of inflammation and its resolution during regeneration, immune cells emerge as central orchestrators of cardiac fate. Translating these fundamental insights into clinical applications represents a major challenge and opportunity for the field. In this Review, we decode the immunological blueprint of heart development and regeneration to transform cardiovascular disease treatment and unlock the regenerative capacity of the human heart.

## 1 Introduction

The heart is a vital organ responsible for sustaining life, forming through a highly coordinated process in early embryogenesis. Its intricate development gives rise to a structure of distinct chambers inter-connected by a complex network of vessels, cells and tissue, all designed to ensure that blood flow, nutrient delivery and oxygenation reach the entire body. Cardiomyocytes are responsible for the contractile function that drives circulation supported by an extracellular matrix scaffold, fibroblasts, endothelial cells and a conduction system that synchronises heartbeats. However, recent insights have highlighted the significant contribution of the immune system to cardiac development and function, an area that has not been comprehensively integrated into our understanding of cardiac biology until now.

Beyond their traditional role in pathogen defence, immune cells emerge as essential architects of cardiac formation. By embryonic day E7.5 in mice, immune cells such as macrophages infiltrate the developing myocardium, embedding themselves within the tissue and becoming integral to the cellular landscape [1]. These early-arriving immune cells establish not only immune surveillance but actively participate in organogenesis. For example, yolk sac-derived macrophages support coronary vasculature formation, establishing the foundation for cardiac blood supply [2]. This developmental contribution exemplifies how immune cells directly shape cardiac development through dynamic interactions with the cardiac niche, influencing growth, structural remodelling and tissue maturation [3–6]. The immune system’s involvement extends beyond development into adult life, where immune cells maintain essential housekeeping roles and orchestrate responses to cardiac insults. Following myocardial infarction, immune cells rapidly infiltrate the damaged heart to clear dead tissue and facilitate healing. However, this response presents a dual nature: while initiating tissue clearance and repair, excessive and prolonged inflammation leads to irreversible tissue damage, contributing to heart failure [7]. In contrast, organisms capable of cardiac regeneration, such as zebrafish, axolotls and the surface-dwelling *Astyanax mexicanus*, demonstrate a highly orchestrated immune response that balances pro-fibrotic and pro-regenerative processes [8–11]. This precise immune modulation enables a transition from initial repair to a regenerative phase, allowing these species to achieve complete cardiac regeneration after injury. Technological advances rapidly expand our understanding of immune-mediated processes in cardiac development and regeneration. Multiomics approaches now enable detailed characterisation of cellular heterogeneity and spatial interactions within complex tissue environments, while next-generation *in vitro* models provide functional insights into immune cell roles [6,12–15]. These tools are transforming descriptive studies into mechanistic understanding of immune function, expanding the frontiers of cardiac immunology.

In this Review, we explore the complex roles of innate and adaptive immune cells in cardiac development and regeneration. We examine how immune cells integrate developmental niches, maintain cardiac homeostasis and orchestrate regenerative responses. We further discuss how recent technological innovations offer deeper insights into these processes, supporting novel therapeutic approaches for addressing congenital heart disease and enhancing regeneration in cardiac patients.

## 2 Immune-mediated cardiac development

**Fig. 1:**
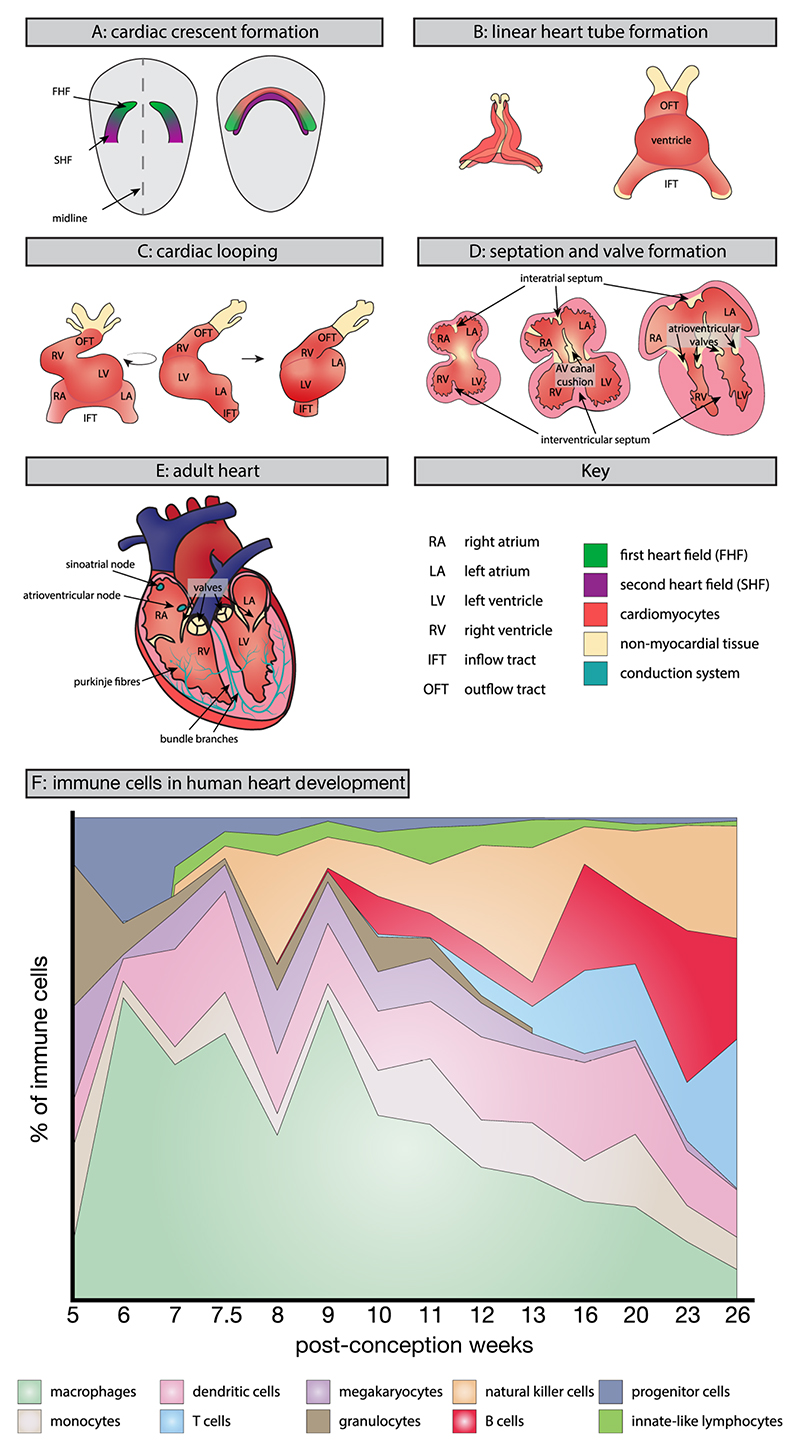
Development of the mammalian heart, illustrations of the major morphological stages of cardiac development. (A) Cardiac progenitors from the first heart field (FHF) and second heart field (SHF) migrate symmetrically along the midline to form the cardiac crescent. (B) The cardiac crescent fuses to create the linear heart tube. (C) Cardiac looping occurs as the heart tube undergoes asymmetric bending and buckling, establishing the helical shape that precedes chamber formation. (D) Chamber formation progresses alongside septation, which divides the heart into distinct regions, while developing valves regulate blood flow direction. (E) These processes result in the mature four-chambered heart, which uses the cardiac conduction system to coordinate rhythmic contractions that circulate blood throughout the body. (F) Immune cells in human cardiac development. Area plot depicting the changing proportions of distinct immune cell populations throughout human cardiac development (5–26 PCW). Macrophages represent the dominant immune population during early development, with later emergence of additional populations including dendritic cells, T cells and B cells [31]. The dynamic shifts in immune cell composition highlight the changing immunological requirements during different developmental stages.

The heart forms as the first functional organ in the developing embryo through a series of precisely coordinated events involving multiple progenitor cell populations, including both cardiac and immune lineages. From early development, immune cells seed the cardiac tissue, with macrophages originating from both yolk sac and foetal liver progenitors (Box 1) to establish diverse developmental functions beyond traditional immune roles. This process integrates developmental and immunological signals to establish a fully functional organ (Fig. 1).

### 2.1 Early heart development

The foundation of cardiac development begins during gastrulation, when pluripotent epiblast cells give rise to mesodermal cells that migrate to the anterior embryo during the second week post-conception (Carnegie stage (CS) 7 in humans) [32–34]. These cells enter a region of Wnt inhibition, which enables cardiac specification, before migrating as two symmetric groups on either side of the midline [35]. Within these migrating mesodermal cardiac progenitors, two subpopulations can be distinguished, the first heart field (FHF) and second heart field (SHF) [33,36]. Signalling pathways, including BMP, FGF and Wnt originating from adjacent endoderm, ectoderm and surrounding tissues, coordinate the fusion of these progenitors into the cardiac crescent (CS8) (Fig. 1A). This process initiates a cardiac-specific transcriptional programme, marked by the expression of Nkx2.5 and Islet1 [37–39]. FHF cells lie within the craniolateral region and form the cardiac crescent, while SHF cells are located more dorsally to the crescent [33,36].

FHF cells swiftly differentiate into cardiomyocytes and even at this early stage, the heart exhibits signs of functionality [40]. Calcium oscillations propagate as waves through the cardiac crescent, playing a critical role in coordinating cardiomyocyte growth and differentiation [40,41]. These waves orchestrate coordinated beating in lateral regions upon sarcomere formation [40]. Cardiac progenitor cells differentiate into both myocardium and endocardium although their lineages are restricted very early in development. These layers are separated by extracellular matrix known as “cardiac jelly” (CS9) [42,43]. Within the FHF, Mesp1^+^ progenitors are unipotent, differentiating exclusively into either cardiomyocytes or endothelial cells. In contrast, the SHF contains additional Mesp1^+^ progenitors, which are bipotent and can later specify into either lineage [36]. Within the heart fields, progenitor sub-populations are already diverging that will contribute to distinct cardiac regions (reviewed in [44]).

By the end of the fourth week of development (CS10), the cardiac crescent fuses along the midline to form a closed heart tube [43] (Fig. 1B). Recent single cell analyses have identified distinct immune populations present at these early stages, including primitive macrophages derived from yolk sac progenitors [6,13]. Studies in Xenopus revealed that the formation of the cardiac tube depends critically on local macrophage populations underlying the myocardium. Their depletion leads to complete failure of the bilateral fields to fuse, high-lighting an essential role for immune cells in mediating early cardiac morphogenesis [45]. These early-arriving immune cells establish themselves as integral components of the developing cardiac tissue, laying the groundwork for subsequent developmental events.

### 2.2 Chamber formation and immune-mediated growth

The heart tube undergoes significant morphological changes as development progresses, coincident with the initiation of a chamber myocardial programme [46,47]. Studies in mice show how progenitor cells from both first and second heart fields contribute to heart tube elongation, enabling increased morphological complexity and functional maturity [48] (reviewed in [49]). The tube develops an asymmetric shape through cardiac looping, bending and buckling while fixed between the venous and arterial poles. Asymmetric pole movement modulates this process, ultimately shaping the tube into a helical form that precedes chamber formation [50] (Fig. 1C). In mice and humans, heart tube elongation and looping coincide with the emergence of a chamber myocardial programme (Nppa and Nppb, Gja5, Smpx/Chisel, Bmp10 and Atp2a2) that specifies the myocardium of primitive chamber regions. Subsequently, the atrial and ventricular chambers balloon out from differentiating myocardium at the inflow and outflow tracts, respectively [34,43,46,47]. Chamber specification occurs under precise molecular control. For example, in mice and humans, Hand1 expression marks FHF-derived myocardial cells of the left ventricle but is also observed in SHF-derived outflow tract cells and the epicardium, high-lighting the need for multiple factors to tightly determine cellular identity [51,52]. This is further exemplified by the T-box family of transcription factors which drive antagonistic gene programmes. Tbx5 and Tbx20 drive the myocardial gene programme essential for chamber formation, while Tbx2 and Tbx3 specify non-chamber regions, including the atrioventricular canal, myocardial venous sinus, outflow tract and the developing conduction system [43,53]. The developing heart then undergoes septation to separate the cardiac chambers, driven in part by Tbx5, which regulates essential Hedgehog signalling networks required for atrial septation [54] (Fig. 1D). Additionally, time-sensitive regulation of Osr1 by Tbx5 in the posterior SHF is crucial for proper cardiac septation [55]. In mammals such as mice and humans, this process ultimately results in the formation of four distinct chambers (two atria and two ventricles) (Fig. 1E), in contrast to other vertebrates such as fish (two chambers) or amphibians and most reptiles (three chambers).

Recent studies have uncovered crucial immune influences during chamber development, beyond previously recognised contributions. Through studies in rats and zebrafish, the IL4/IL4R-STAT3 pathway has emerged as a key regulator of cardiomyocyte proliferation, driving the expression of cell cycle progression genes such as c-myc, cyclin d1, and gata3 [56,57]. Similarly, mouse models reveal that IL13 signalling regulates cardiomyocyte cell division in the developing myocardium. Hearts deficient in IL13 exhibit reduced cardiomyocyte cell cycle activity and altered expression of growth-related genes [5]. Murine studies show the significant impact of maternal regulatory T cells on cardiomyocyte proliferation. Depletion of these cells leads to reduced cardiomyocyte proliferation, revealing an unexpected link between maternal immunity and foetal cardiac growth [3]. Furthermore, recent multiomic analyses of the developing human heart have identified novel immune-dependent pathways involved in chamber maturation, suggesting a broader and more intricate role for the immune system in cardiac chamber development than previously understood [6]. In fact, the dynamic shifts in immune cell composition during different developmental stages of the human heart highlight the changing immunological requirements as development progresses (Fig. 1F).

### 2.3 Valve development and remodelling

In birds and mammals, the process of cardiac chamber separation begins with the development of four major endocardial cushions: two in the atrioventricular canal (AVC) and two in the outflow tract. These cushions, consisting of expanded cardiac jelly between the endocardial and myocardial layers, initiate valve development around E9.5 in mice. Endocardial cells undergo endothelial-to-mesenchymal transition (EndoMT), forming thick primitive valves that undergo extensive remodelling to achieve their mature structure. This remodelling transforms endocardial cushions into distinct valve structures: the mitral and tricuspid valves develop from the major atrioventricular cushions (septal leaflets) and minor lateral cushions (free wall leaflets), while the semilunar valves of the aorta and pulmonary trunk arise from the major outflow tract cushions and intercalated ridges (reviewed in [58]).

**Fig. 2:**
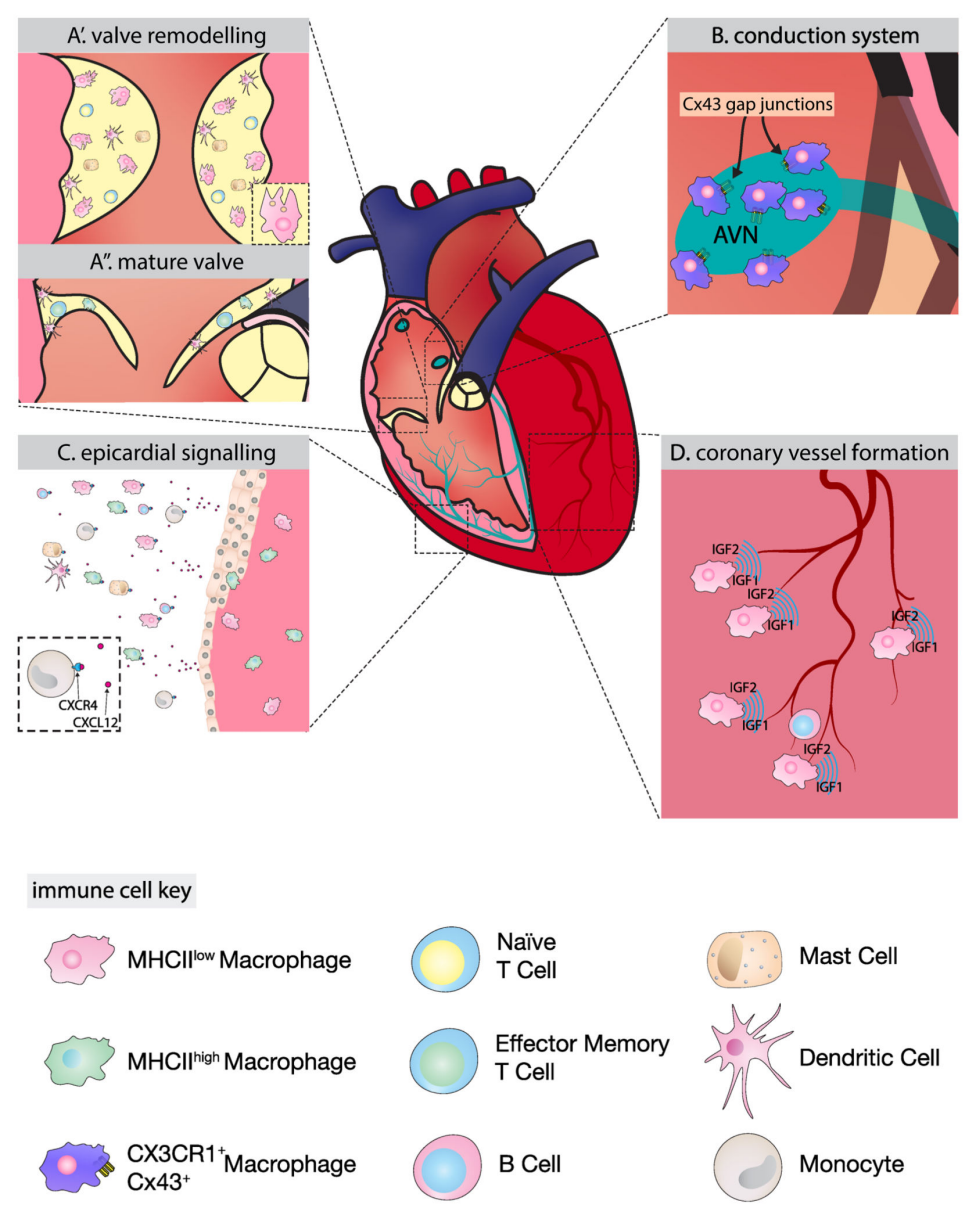
Specialised immune niches in the developing heart. (A) The valve immune niche undergoes distinct transitions during development. During active remodelling (A’), the niche comprises naïve T cells, dendritic cells, mast cells and MHCII^low^ phagocytic macrophages. As valves mature (A”), the immune population shifts toward an antigen-presenting profile with dendritic cells, effector memory T cells and MHCII^high^ macrophages. (B) In the conduction system, CX3CR1^+^ macrophages establish direct connections with cardiomyocytes through Connexin 43 gap junctions within the atrioventricular node (AVN), enabling coordinated electrical impulse propagation. (C) The epicardial niche functions as a recruitment hub, where epicardial-derived CXCL12 attracts CXCR4-expressing immune cells to establish the subepicardial immune compartment. (D) Within the developing coronary vasculature, MHCII^low^ macrophages secrete IGF1 and IGF2 to promote vessel formation, while B cells interact directly with the developing endothelium to support vascular development.

Single cell RNA sequencing of cardiac valves during murine postnatal remodelling (P7 to P30) identified five immune cell populations: T cells, dendritic cells, mast cells, Mrc1^+^ macrophages and CCL2^+^ CCL4^+^ macrophages. During this period, Mrc1^+^ macrophages, which express differentiation markers such as Dab2, Maf and Csf1r, along with mast cells and T cells, decline in number. Furthermore, T cells also shift from a naïve to an effector memory phenotype. Meanwhile, CCL2^+^ CCL4^+^ macrophages express chemokines such as Tnf and Cd74. These findings suggest that as the valves mature, the immune niche transitions from a predominance of Csf1r^+^ macrophages towards a composition dominated by antigen-presenting macrophages, potentially reflecting a shift in the immunological requirements of the developing valves [59]. The role of T cells and mast cells in valve development remains unclear, but macrophages play a pivotal role in valvular remodelling. A specialised population of highly phagocytic cardiac resident macrophages orchestrates this process, transforming the endocardial cushions into mature valve structures [4] (Fig. 2A). These macrophages were reported to originate from the endocardium around E10.5, based on Nfatc1 lineage-tracing experiments in mice, suggesting transient local haematopoiesis as the source of these cardiac resident macrophages [4]. This finding challenges the prevailing view that all embryonic macrophages derive exclusively from the yolk sac and foetal liver ([1,60–64], Box 1 and Box 2).

### 2.4 Cardiac conduction system

The cardiac conduction system is a critical component of the heart, responsible for coordinating the rhythmic contractions of the cardiac muscle. It develops through precise patterning of specialised cardiac cells and comprises slow-conducting nodes (sinoatrial and atrioventricular nodes) and fast-conducting components (atrioventricular bundle, Purkinje fibres and bundle branches) (reviewed in [68]). In the developing mouse heart tube, progenitors committed to the sinus venosus and sinoatrial node (SAN) are marked by the Tbx18^+^, Isl1^+^ and Nkx2.5^+^ transcriptional signature [69]. The atrioventricular canal forms in the centre of the developing heart, driven by Bmp2 expression and subsequent activation of Tbx2 and Tbx3 transcription factors that suppress the myocardial programme [70–72]. The Purkinje fibre network emerges in stages, initiated by precursors committed early in development, followed by a later phase of rapid recruitment of bipotent progenitors from Cx40^+^ trabeculae, with further development occurring postnatally [73–76]. The proper development of the cardiac conduction system is essential to drive the circulation of blood through the body. Macrophages play essential roles throughout the development and maturation of the conduction system. Recent studies have identified specific macrophage populations and immune signalling axes within the developing human conduction system [6], suggesting their involvement in establishing proper electrical conduction patterns. A microglia-like subset of CX3CR1^+^ macrophages has been localised within the developing SAN. Multiomic analyses revealed that these macrophages are recruited via CX3CL1 expressed in the SAN. Most abundant during the first trimester of development, these macrophages interact with the pacemaking niche via signalling molecules characteristic of neuronal homeostasis, including IGF1-IGF1R, CD200-CD200R1, CD47-SIRPA, P2RY12 and TREM2. These findings strongly suggest that macrophages play a regulatory role in the parasympathetic innervation of the SAN [6]. Their role in regulating cardiac rhythm in the homeostatic mouse heart further supports their importance during development [77] (Fig. 2B). Although the detailed mechanisms of immune-mediated regulation during conduction system maturation remain unclear, the abundance of specialised immune populations in the myocardium, including macrophages, B cells and NK cells, during postnatal Purkinje network remodelling suggests ongoing immune involvement in this process [78]. Recent work also identified immune cells within the neonatal ventricular conduction system and suggested that inflammatory factors may influence connexin expression during the critical postnatal window when regenerative capacity transitions to a pro-fibrotic response [79].

### 2.5 Epicardial-immune cell interactions and vascular development

The epicardium is an essential coordinator of immune cell recruitment and cardiac development. It originates from the pro-epicardium at the inflow tract base during the fourth week post-conception (human CS11) [80]. Lineage tracing studies in mice identified a novel cardiac progenitor pool marked by the expression of Mab21l2, which contributes to the pro-epicardium at E9.5 and represents the earliest epicardial progenitors [39]. As an epithelial-like layer, the epicardium serves as a critical signalling centre, promoting cardiomyocyte proliferation and chamber growth, while contributing epicardial-derived cells (EPDCs) to the developing heart [81–84]. By the fifth week (CS15), the epicardium completely envelops the heart and expands into multiple layers in ventricular regions (CS17) [85]. During the eighth week (CS23), EPDCs expressing Zeb1 and Vimentin undergo epithelial-to-mesenchymal transition (EMT), contributing to cardiac fibroblast populations and coronary vessel development [85].

The epicardium also directs the recruitment of immune cells. Specific epicardial populations actively attract CD45^+^ leukocytes and primitive yolk sac macrophages (MHCII^low^) to the developing heart [86,87] (Fig. 2C). Within the developing zebrafish epicardium, an epicardial subpopulation strongly expresses the chemoattractant cxcl12a, which diffuses into the surrounding pericardial cavity and drives the recruitment of CD45^+^ leukocytes and myeloid cells expressing the cognate receptor cxcr4b [86]. Similarly, in mice lineage tracing studies have identified CD45^+^ haematopoietic cells in the developing epicardium originating from both primitive and definitive haematopoiesis [88]. Around E11.5–12.5, yolk-sac macrophages seed the subepicardial space and play a crucial role in vascular development [89] (Fig. 2D). Consequently, ablation of the epicardium and knockout of the epicardial transcription factor WT1 lead to abnormal coronary vessel development, highlighting the importance of epicardial-immune cell interactions in cardiovascular development [87].

The coronary vasculature is critical to the maintenance of cardiac function, shuttling oxygen and nutrients throughout this hard-working organ. Therefore, the development of the vascular network is carefully regulated by specialised immune populations. Distinct macrophage populations orchestrate different aspects of vascular development. CCR2^-^ yolk sac macrophages promote coronary artery development through IGF1 and IGF2 signalling, and macrophage-deficient mice (Csf1-dependent) exhibit abnormal coronary branching [2]. Lyve1^+^ cardiac resident macrophages localise at vessel branching points around E14.5 and guide lymphatic vessel formation through hyaluronan-mediated interactions [89]. While CCR2^+^ macrophages (MHC-II^high^) from foetal liver progenitors are dispensable before E14.5, their potential roles in later development require further investigation [87,89]. During human embryonic development, 89 % of cardiac macrophages are either transitioning to a proangiogenic state or expressing a proangiogenic signature, including high levels of IL1B, VEGFA, CXCL8, TNF, BTG1, and SOD2. These macrophages preferentially localise to perivascular niches [31].

B lymphocytes also play unexpected roles in vascular development. A specific population of follicular naive B cells contacts the microvascular endothelium, with a subset transitioning into the myocardium. These cells are crucial for normal cardiac development, as B cell deficiency results in reduced myocardial mass, due to smaller cardiomyocytes, and altered left ventricular function [90].

## 3 Immune cell populations in cardiac homeostasis

The developmental seeding of immune populations in the heart creates specialised tissue resident networks that persist into adulthood. These populations maintain distinct spatial organisations and functions essential for cardiac homeostasis, whilst retaining the capacity to respond to injury and stress. Studies in mice reveal that myeloid cells dominate the homeostatic cardiac immune landscape, comprising more than 80 % of cardiac leukocytes and 7–10 % of the nonmyocyte compartment [91].

### 3.1 Macrophage distribution and function

Cardiac macrophages maintain distinct spatial distributions reflecting their developmental origins. This compartmentalisation supports diverse homeostatic functions, from traditional immune surveillance and debris clearance to tissue-specific roles [92] (reviewed in [93]). In zebrafish, differential abundance patterns were revealed by analysis of transgenic reporters for csf1ra and mpeg1.1. While both populations predominate in the ventricle during development, adult hearts show more complex organisation. Primitive-derived csf1ra^+^ mpeg1.1^-^ macrophages mainly occupy the atrial surface, while csf1ra^-^ mpeg1.1^+^ and csf1ra^+^ mpeg1.1^+^ macrophages distribute throughout the myocardium [94]. Similar differential distribution patterns exist in mice, where macrophages expressing CD45^+^ CD11b^+^ F4/80^+^ are distributed in the myocardium adjacent to cardiomyocytes, playing a crucial role in clearing damaged mitochondria. Damaged mitochondria accumulate in cardiomyocytes and are expelled in exophers formed via the autophagy machinery. Exophers are taken up and degraded by these macrophages via expression of phosphatidylserine, which is recognised by Mertk-expressing cardiac macrophages [93,95,96]. Beyond mitochondrial clearance, another example of macrophages establishing essential interactions with cardiomyocytes is in the mammalian conduction system, where macrophages coordinate electrical impulse propagation through Connexin 43 gap junctions [77]. These interactions are also observed in the human heart, where spatial transcriptomics revealed macrophage presence in both sinoatrial (SAN) and atrioventricular (AVN) nodes. The SAN shows distinct compartmentalisation, with a central region containing pacemaker cells, fibroblasts and glial cells, surrounded by a peripheral region enriched with LYVE1^+^ macrophages that express TGFβ1, suggesting a role in ECM homeostasis through interaction with TGFβR1-expressing fibroblasts [12]. Additionally, single cell analysis of human cardiac tissue has also identified cardiac-specific LYVE1^+^ and antigen-presenting macrophages [97]. Outside the myocardium itself, the pericardial cavity harbours a population of macrophages that are phenotypically distinct from cardiac resident macrophages but share transcriptional similarities with macrophages in peritoneal and pleural cavities. These CD11b^+^ MHCII^-^ ICAM2^+^ F4/80^+^ GATA6^+^ macrophages are embryonically derived and later replenished by monocyte-derived macrophages, having been found in mice, humans and pigs [98]. Although their homeostatic role remains unclear, expression of lubricin suggests a potential contribution to pericardial lubrication [99,100].

### 3.2 Dendritic cells

Dendritic cells (DCs) maintain surveillance networks throughout the heart, serving dual roles in innate and adaptive immunity. These antigen-presenting cells phagocytose and process nearby antigens, comprising three distinct populations: classical DCs, plasmacytoid DCs and inflammation-induced monocyte-derived DCs (reviewed in [101]). In murine hearts, DCs constitute 1 % of cardiac leukocytes, with classical DCs marked by high zbtb46 expression predominating [1,102]. Single cell heart atlases have also identified plasmacytoid DCs in the adult human heart [97]. Unlike tissue resident macrophages, cardiac DCs undergo continuous replacement by circulating precursors, though maintaining limited *in situ* division capacity and passing their antigen cross-presentation repertoire to daughter cells [103]. Studies in mice reveal that these cells concentrate in regions of turbulent flow, particularly cardiac valves, extending processes into both cardiac interstitium and vessel lumens to sample antigens from tissue and circulation [104].

### 3.3 Mast cell distribution and function

Mast cells transition from predominantly yolk sac-derived populations during development to bone marrow-derived cells in adulthood. In mice and humans, these granulocytes, crucial for both inflammation and allergic responses [105,106], establish specific distribution patterns throughout cardiac layers, with highest density in the epicardium while also populating myocardium and endocardium [107, 108]. Single cell sequencing studies in mice revealed developmental yolk sac-derived mast cells express genes governing vascular and nerve patterning, including EphA2, PlxnC1 and Tac1 [109,110]. Adult distribution patterns reflect this developmental signature, with 31 % of myocardial mast cells maintaining perivascular positions, suggesting ongoing crosstalk with nervous and vascular systems [108].

### 3.4 The epicardial immune niche

The epicardium acts as an immune hub, integrating multiple immune populations including developmentally-seeded macrophages and mast cells [87,94,108]. Recent single cell and nuclear RNA sequencing analyses of foetal (6–10 post-conception weeks) versus adult human hearts reveal a developmental transition in epicardial function. The epicardium shifts from promoting proliferation, angiogenesis and EMT to maintaining an immune-response primed state in adulthood [111]. This shift in function is accompanied by changes in the organisation and composition of the epicardial immune niche [12]. Multiomics analyses of the adult human heart revealed precise organisation of immune and stromal populations forming a pre-primed defensive barrier. LYVE^+^ IGF1^+^ resident macrophages distribute throughout the epicardium and sub-epicardium of all chambers. These macrophages, along with fibroblasts, endothelial and epicardial cells, orchestrate immune recruitment through CCL2 and CXCL12 signalling, particularly affecting plasma B cells expressing CCR2 and CXCR4. B cells show remarkable spatial organisation, with distinct IgG and IgA-expressing territories. Lymphatic endothelial cells recruit IgA^+^ plasma B cells via CCL28-CCR10 signalling, while macrophages support B cell survival through BAFF/TNFSF13B expression and co-localisation with TNFRSF13B^+^ B cells. Within this niche, cells interact via TGFβ signalling, suggesting involvement in ECM regulation [12]. Together, these findings highlight the complex organisation and function of the epicardial immune niche in maintaining cardiac homeostasis and defence in the adult heart.

## 4 Immune orchestration of cardiac repair and regeneration

The transition from homeostatic maintenance to injury response requires precise coordination of immune populations within the heart. While all vertebrates initiate similar immediate responses to cardiac injury, the ultimate outcome of repair versus regeneration depends on species-specific immune modulation of the healing process.

### 4.1 Initial immune responses to cardiac injury

Following myocardial infarction, coronary artery occlusion deprives cardiac tissue of oxygen, triggering cellular death and an immediate immune cascade. The initial response begins with resident cardiac cells: mast cells degranulate to release pre-formed TNFα, histamine and proteases, while cardiomyocytes and resident macrophages produce inflammatory cytokines and chemokines [112–116]. This inflammatory milieu recruits neutrophils and monocytes from circulation to clear necrotic tissue.

The subsequent healing response diverges between species. In adult mammals, the inflammatory phase transitions to a reparative phase as inflammatory mediators decline. This transition is orchestrated by CCR2^+^ Ly6C^low^ monocyte-derived macrophages, which emerge from pre-existing inflammatory Ly6C^high^ monocyte-derived macrophages [117,118]. These reparative macrophages secrete TGFβ, activating resident fibroblasts into collagen-producing myofibroblasts, and directly produce collagen and VEGF, promoting scar formation and endothelial cell proliferation [8,119,118]. Despite such coordinated repair, the process leads to a permanent, non-contractile fibrotic scar. When injury is extensive or immune responses become dysregulated, progressive ventricular remodelling leads to heart failure [7]. In contrast, neonatal mammals and certain vertebrates including zebrafish, salamanders, axolotls, xenopus and surface-dwelling *Astyanax mexicanus* maintain regenerative capacity. While these species initiate similar inflammatory and reparative responses, they progress beyond scarring to achieve complete regeneration through precisely controlled immune modulation (reviewed in [120]).

### 4.2 Immune-mediated regeneration: from scar formation to resolution

**Fig. 3:**
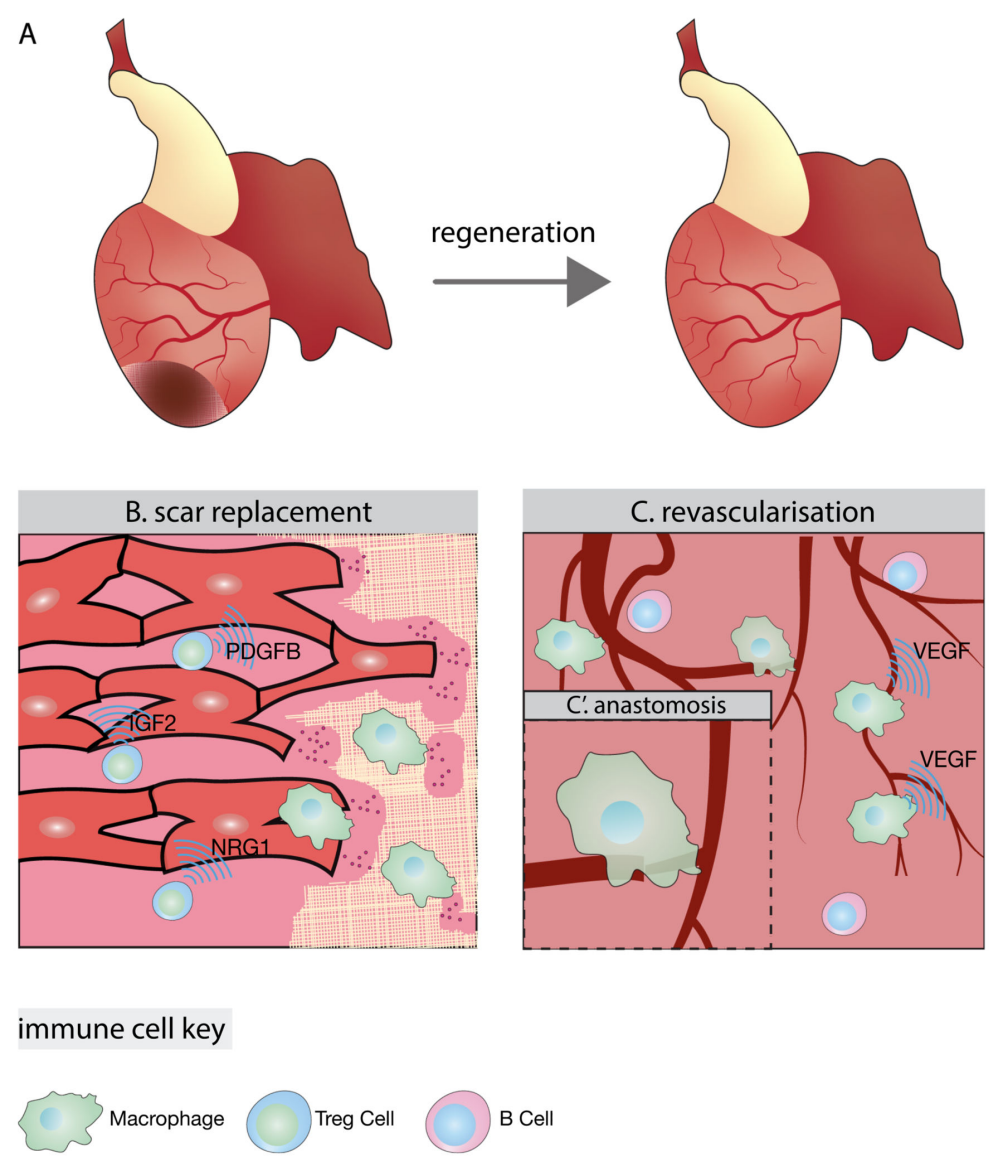
Immune orchestration of cardiac regeneration. (A) Overview of the regenerative response in species such as zebrafish, where a temporary scar (dark region) is progressively replaced by functional cardiac tissue through immune-mediated processes. (B) At the injury border zone, immune cells coordinate scar replacement through multiple mechanisms. Macrophages facilitate cardiomyocyte invasion into the fibrotic region, while regulatory T cells (Tregs) directly contact cardiomyocytes and secrete regenerative factors including PDGFβ, IGF2 and NRG1. (C) The revascularisation process requires precise immune-vascular coordination. Macrophages promote vessel growth through VEGF secretion. (C′) Detailed view of vessel anastomosis, where macrophages directly facilitate the fusion of endothelial tip cells, recapitulating their developmental role in vascular patterning.

Regeneration requires precisely coordinated immune responses rather than simply avoiding inflammation. In regenerative species, immune cells orchestrate a ‘patch and replace’ strategy, where initial scar formation provides temporary structural support before complete regeneration occurs. This process depends on finely tuned temporal balance of immune cell subsets, each playing specific and essential roles (Fig. 3).

In the initial days post-injury, pro-inflammatory macrophages expressing IL1β, TNFα and IL6 accumulate at injury sites [118]. Beyond clearing debris and death tissue, in both zebrafish and mice, these cells then transition to an anti-inflammatory phase expressing IL10, TGFβ and VEGF, interacting with cardiac fibroblasts and directly participating in extracellular matrix deposition [8,9,118,121]. The timing of macrophage responses proves critical, as their depletion in salamanders leads to abnormal early fibroblast activation and increased LOX enzyme expression, resulting in highly cross-linked permanent scars [10]. Comparative studies between zebrafish and the non-regenerative medaka reveal that even delayed macrophage arrival impairs regeneration [122], highlighting the critical timing of immune responses.

The transition to regeneration involves coordinated immune-mediated tissue remodelling. At the injury border zone in zebrafish, macrophages establish direct interactions with cardiomyocyte protrusions, cooperatively producing MMP14b metalloproteinases to degrade extracellular matrix. This remodelling allows cardiomyocyte invasion into scarred regions [123] (Fig. 3B). Transcriptomic studies in zebrafish revealed specialised macrophage populations that facilitate regeneration, including two populations marked by hbaa and timp4.3 expression that expand specifically during regeneration. Their shared CCR2^low^ signature suggests primitive origins [1,124]. Following injury, these populations seem to take on distinct functions, with hbaa^+^ macrophages alleviating oxidative stress through hmox1a expression, while timp4.3^+^ macrophages contributing to ECM remodelling. Notably, ablation studies show that circulating monocyte-derived macrophages cannot compensate for the loss of primitive-derived macrophages, resulting in impaired regeneration [124]. This highlights the specialised tissue functions unique to cardiac resident macrophages, which appear to possess regenerative capabilities that infiltrating monocyte-derived cells lack. Unlike infiltrating monocytes, which often promote inflammatory responses and fibrosis, tissue-resident macrophages maintain tissue integrity through their specialised developmental programming. Additionally, recruited wt1b^+^ macrophages contribute to tissue restoration by persisting beyond the inflammatory phase and coordinating ECM remodelling through expression of timp2b and mmp14a [125].

Successful regeneration requires rapid revascularisation, achieved through immune-vascular interactions. In zebrafish, revascularisation begins hours after injury, involving both superficial and deep vessel formation. Hypoxia drives superficial revascularisation through HIF1α-mediated epicardial cxcl12b expression, while endocardial VEGFA signalling promotes intraventricular vessel sprouting [126,127]. In mice, CCR2^+^ Ly6C^low^ macrophages facilitate this process through VEGF secretion and direct participation in the fusion of endothelial tip cells via vessel anastomosis (Fig. 3C) [118,128]. The function of these macrophage populations critically depends on the local environment. This is demonstrated through adoptive transfer experiments in both zebrafish and mice, where macrophages from non-scarring settings promote excessive fibrosis when placed in scar-prone environments [8]. Similarly, the MyD88 pathway exemplifies context-dependent immune regulation. Its activation proves essential for controlled inflammation in regenerative species but exacerbates damage in non-regenerative mammals [129,130].

The regulation of cardiac regeneration is age-dependent. While adult mammals form permanent scars after cardiac injury, neonatal hearts can achieve complete regeneration, similar to adult zebrafish and sala-manders. In mice, this regenerative capacity persists for the first week of life, coinciding with unique immune characteristics [131]. At postnatal day 1 (P1), cardiac injury responses rely primarily on tissue resident macrophages, with minimal monocyte infiltration from circulation. By postnatal day 7 (P7), this pattern shifts dramatically, with hearts showing significant monocyte infiltration. The transition from tissue resident macrophage-dominated response to monocyte infiltration marks a critical switch from regenerative to fibrotic healing. Tissue resident macrophages appear to coordinate regenerative programmes through their specialised developmental programming and tissue-specific functions, whereas the infiltrating monocyte-derived macrophages tend to promote inflammatory responses leading to fibrosis and scarring. Macrophage depletion experiments at P1 result in failed regeneration and fibrotic scarring [131], demonstrating how precisely regulated immune responses, not just cardiomyocyte proliferative capacity, determine regenerative success. These experiments highlight the critical interdependence between immune cells and cardiomyocytes, with successful regeneration requiring both appropriate immune orchestration and cardiomyocyte proliferative potential. While immune cells provide necessary support by creating permissive conditions, they cannot drive regeneration without responsive cardiomyocytes capable of dedifferentiation and proliferation. Neither component alone is sufficient. The age-dependent regenerative ability may also relate with adaptive immune responses and how they interact with innate immune cells to further modulate regeneration.

Adaptive immune responses add further complexity to regenerative regulation. Regulatory T cells demonstrate conserved pro-regenerative functions across species. In zebrafish, Foxp3^+^ Treg-like cells rapidly migrate to damaged organs, peaking at 7 days post injury and persisting throughout regeneration [132]. These cells directly contact proliferating cardiomyocytes and secrete tissue-specific regenerative factors including Nrg1, IGF2 and PDGFB. Both Treg-derived factors and Foxp3 expression prove essential, as either Treg ablation or Foxp3 knockout significantly impairs regeneration [132]. This regenerative role extends to mice, where CD4^+^ FOXP4^+^ Tregs promote cardiomyocyte proliferation in both neonatal and adult stages through secretion of CCL24, GAS6 and AREG [3,133].

In contrast, CD4^+^ T cells, elevated in non-regenerative P8 mice, inhibit regeneration through release of TNFα, IFNγ and IL17A, characteristic of Th1 and Th17 subtypes, thereby suppressing cardiomyocyte proliferation. Accordingly, CD4^+^ T cell ablation enables limited extension of neonatal regenerative programmes beyond P7 [134]. Conversely, cardiac B cells support regeneration in neonatal mice through anti-inflammatory and pro-angiogenic functions, though cardioprotective S100a6^high^ and S100a4^high^ clusters diminish in adult hearts (Fig. 3C) [135].

Recent zebrafish studies identified MHC class II antigen presentation signatures (mhc2a, cd74a, cd74b) in macrophages, B cells and a distinct subpopulation of activated endocardial cells expressing immune cell-like markers. CD4^+^ T cells localise proximal to these endocardial cells, and genetic blockade of MHC class II antigen presentation impairs both CD4^+^ T cell and endocardial cell infiltration, reducing cardiomyocyte dedifferentiation and proliferation [136]. The conservation of cd74 expression in neonatal mouse endocardial cells post-injury suggests potential evolutionary conservation of this mechanism [136]. Recent work suggests that injury to the zebrafish heart may activate haemogenic activity in the endocardium, although the precise contribution of these endocardium-derived leukocytes to regeneration requires further study [137].

This intricate immune orchestration of cardiac regeneration demonstrates how successful tissue restoration requires precise temporal control of both pro-inflammatory and pro-regenerative signals. Understanding these carefully balanced immune responses may provide crucial insights for therapeutic approaches aimed at enhancing regeneration in non-regenerative species.

## 5 Immune cell dynamics in developmental and regenerative cardiac niches

The formation and remodelling of immune cell niches are crucial in both cardiac development and regeneration, though their organisation and function adapt to distinct physiological demands. During development, immune cells establish specialised compartments that lay the foundation for adult cardiac function, whilst regenerative niches must orchestrate both temporary repair and tissue restoration.

During early cardiac development, interactions between immune cells and forming cardiac tissues begin in the heart tube, where macrophages guide morphogenesis [45]. As cardiac chambers emerge, immune-myocardial niches form, with IL4/IL13 signalling and maternal Treg cells influencing cardiomyocyte proliferation [3,5,56]. The developing valves establish complex immune niches where specialised macrophages coordinate remodelling, alongside T cells and mast cells [4, 59]. Within the conduction system, CX3CR1^+^ macrophages create a unique niche in the sinoatrial node, interacting with the pacemaking environment through neuronal homeostasis signals [6] and influencing electrical patterning [77]. Perhaps the most intricate developmental niche forms in the epicardium, where distinct macrophage populations (CCR2^-^ and Lyve1^+^) differentially regulate coronary and lymphatic vessel formation, respectively [2,89].

Regenerative niches, while sharing some features with developmental counterparts, must rapidly transition between inflammatory and reparative states. The initial injury niche contains pro-inflammatory macrophages expressing IL1β, TNFα and IL6, which both clear debris and initiate repair [118]. At injury borders, specialised hbaa^+^ and timp4.3^+^ macrophage populations establish regeneration-specific domains, interacting with cardiomyocytes to degrade scar tissue, a process absent in development [123,124]. The vascular regeneration niche recruits CCR2^+^ macrophage populations for vessel anastomosis, whereas developmental vessel formation relies on CCR2 - macrophages [126,128], with both contexts requiring precise immune-endothelial coordination.

The influence of the local environment is crucial in both settings but operates differently. Developmental niches respond to pre-established morphogen gradients and tissue-specific signals, creating stable specialised domains. Regenerative niches must instead rapidly shift from inflammatory to pro-regenerative states, demonstrated dramatically by adoptive transfer experiments where environmental cues override intrinsic macrophage programming [8]. This environmental dependency becomes particularly evident in the age-dependent loss of regenerative capacity, where neonatal hearts maintain developmental-like immune responses, but adult hearts shift toward pro-fibrotic programmes [131]. However, while developmental niches are increasingly well characterised, the precise nature of regenerative niches, including their cellular composition, molecular signatures and spatial organisation, remains largely underexplored. A deeper understanding of these regenerative microenvironments may prove crucial for identifying factors that programme immune cells toward regeneration rather than fibrosis, potentially informing therapeutic strategies to enhance cardiac repair.

## 6 Technological and analytical advances in cardiac immunology

Recent technological breakthroughs provide unprecedented insights into immune cell function during cardiac development and regeneration. Large-scale efforts, including the Human Heart Cell Atlas, demonstrate how single cell and spatial technologies uncover previously unrecognised immune cell diversity [6,12,97,138–140]. These approaches, combined with advanced computational analysis and *in silico* modelling, uncover distinct immune subpopulations and their molecular programmes across developmental stages, homeostatic conditions and injury responses. Multi-modal analyses now enable detailed study of immune cell behaviour within specific cardiac niches. From early developmental seeding to adult tissue maintenance and repair, these technologies reveal how immune cells establish specialised microenvironments and adapt their functions to local tissue needs. Such insights create opportunities to dissect the complex array of immune cell states and their molecular regulation, advancing our understanding of cardiac biology, while suggesting new therapeutic approaches for both congenital and acquired heart conditions.

### 6.1 Single cell technologies uncover diversity of immune cell programmes

Immune cells demonstrate remarkable plasticity, adapting their functions based on local environment and developmental context. While macrophages were traditionally categorised into inflammatory and reparative phenotypes, single cell RNA sequencing has revealed unprecedented heterogeneity in cardiac immune populations, with distinct transcriptional programmes influencing development, homeostasis and regeneration [93,141,16,142]. This heterogeneity becomes particularly evident following myocardial infarction [142]. Spatiotemporal analysis reveals dynamic shifts in macrophage populations in mice: Lyve1^+^ Folr2^+^ tissue-resident macrophages dominate the homeostatic heart but diminish immediately post-injury, only to reappear during later repair stages. Meanwhile, CCR2^+^ Chil3^+^ pro-inflammatory monocyte-derived macrophages are recruited during early responses, peaking at day one post-MI before declining [142]. Beyond transcriptional diversity, immune cells show heterogeneity at epigenetic and metabolic levels. Technologies like scATAC-seq, CUT&RUN and CUT&TAG reveal how chromatin accessibility and transcription factor binding regulate immune cell identity and function. Integration of these data enables construction of gene regulatory networks that define cell states and predict highly regulated responses to injury. These multi-modal analyses require sophisticated computational approaches. Advances in computational modelling allows integration of these modalities, building gene regulatory network (GRN) models [143–145]. GRNs define the transcriptional activity and function of a cell [146]. The expression of a transcription factor (TF), as well as interaction with regulatory regions in the genome are essential information to model a GRN, in combination with the target gene expression of TFs. Several computational approaches exist to model GRNs and they have been discussed extensively in the past [146–149]. Studies in zebrafish hearts demonstrate the power of this multi-modal approach [86,150]. Analysis of chromatin accessibility and transcriptomics identified how inflammatory macrophages signal to activate epicardial fibroblasts and endocardial cells through AP-1 transcription factors, coordinating matrix remodelling, angiogenesis and cardiomyocyte proliferation. Identification of specific enhancer elements controlling these processes suggests potential targets for promoting regeneration over fibrosis [150].

### 6.2 Spatial technologies define immune cell niches and interactions

While single cell analyses identify distinct immune populations, understanding their function requires spatial context within the developing and adult heart. Cells do not function in isolation; rather, their cellular state, behaviour and molecular programmes are shaped by the niche or microenvironment they reside in. Although traditional approaches using fluorescent antibodies and reporter lines revealed broad immune cell distributions, these methods prove time consuming and inherently limited, as they provide information on only a small number of markers, often insufficient to capture the full cellular heterogeneity within a lineage. New spatial ‘omics technologies now address the critical need for mapping cellular heterogeneity within intact tissues (reviewed in [151]). Platforms like the 10X Visium have uncovered previously unknown immune niches in human hearts. Tissue resident macrophages establish distinct territories within the human epicardium and sinoatrial node, with epicardial populations expressing CCL2, CXCL12 and TNFSF13B to interact with local B cells [12,152]. While Visium and GeoMx operate as a spot-based technology, capturing small areas of the tissue with gaps between spots, more high-resolution technologies like Visium HD and Stereo-seq enable near-cellular level resolution at a whole transcriptome level [153–156]. Even though sequencing and spot-based spatial technologies offer a comprehensive whole-transcriptome view, their relatively coarse resolution can make it difficult to study small cell types and developing organs in detail. In contrast, multiplexed imaging-based technologies including, but not limited to, MERSCOPE, Xenium and CosMx enable single-molecule RNA detection at subcellular resolution, though with limited panel sizes [157–159]. These advancements allow identification of rare cell types and distinct niche structures across complex cardiac tissue, placing heterogeneous immune populations in their precise cellular context [160,161]. Complementary spatial proteomics approaches through Imaging Mass Cytometry (IMC) and Multiplexed Ion Beam Imaging by Time of Flight (MIBI-TOF) add protein-level detail by simultaneously detecting over 40 markers *in situ*. However, significant challenges remain. Besides high costs, these technologies lack extensive bench-marking of capture rates and performance (reviewed in [162–164]). Documentation and optimisation protocols for different tissues require standardisation. Furthermore, computational pipelines struggle with precise cell segmentation, especially for non-round and irregularly shaped cells like epithelial cells, polynucleated cardiomyocytes and activated fibroblasts. Current fluorescence-based technologies also face limitations in gene panel design, potentially biasing detection toward specific cell types or activation states. Nevertheless, spatial technologies are transforming our understanding of immune-cardiac interactions, enabling comparisons of cellular context between disease and homeostasis, during development, and across organs and species.

### 6.3 Multi-modal data integration and in silico modelling

The complexity and heterogeneity of immune cell dynamics during development and regeneration requires computational approaches to integrate multiple data modalities. Beyond describing expression patterns and cell populations, these datasets enable modelling of the molecular programmes driving immune cell behaviour. Recent advances in computational modelling allow integration of these modalities to construct predictive GRNs that define cell states and functions. Integration of multimodal data, such as scATAC-seq and scRNA-seq, reveals how TFs interact with regulatory genome regions to control immune cell states. Tools like SCENIC+ [143] and CellOracle [144] enable prediction of regulatory programmes and *in silico* modelling of gene perturbation. For example, analysis of cardiac injury responses identified RUNX1 as a key regulator of recovery [165]. Computational modelling predicted that its deletion would transition macrophages and fibroblasts toward recovery-associated phenotypes. Beyond single cell analysis, cell-cell communication modelling provides crucial insight into immune-cardiac interactions. Platforms like NicheNet [166] and MultiNicheNet [167] predict not only ligand-receptor interactions but also downstream signalling and target gene expression. When combined with spatial data, tools like NicheCompass [168] enable identification of specific niches within cardiac tissue, allowing refined modelling of cellular communication networks.

The integration of these computational approaches provides unprecedented insight into cardiac immunology. By combining single cell analysis, spatial mapping and network modelling, we can now identify specialised immune populations within specific cardiac niches, track their state transitions during development and regeneration, and predict molecular regulators of their function. Such comprehensive understanding suggests new therapeutic targets and enables modelling of the complex immune-cardiac interactions that govern heart development, homeostasis and repair.

### 6.4 In vitro systems to model cardiac-immune interactions

*In vitro* models provide essential platforms for investigating cardiacimmune interactions in developmental and disease studies. Most organs have tissue resident immune cells, yet many 3D *in vitro* models lack this crucial component, limiting their ability to reproduce steady-state and disease processes [169]. Among various immune cells, macrophages have received particular attention due to their ubiquitous presence and essential role in maintaining organ function [17].

Traditional 2D co-cultures have provided fundamental insights into interactions between macrophages and cardiomyocytes, revealing macrophages as key supporters of cardiac function rather than merely innate immune cells [77]. Recent studies have shown that co-culturing hiPSC-derived macrophages with cardiomyocytes enhances maturation marker expression and facilitates synchronous beating through electrical coupling [170]. However, 2D systems cannot capture the complex three-dimensional organisation of cardiac tissue and its specialised niches. More advanced 3D cultures address these limitations by integrating multiple cell types, better mimicking the heart’s cellular composition and improving physiological relevance. Human engineered cardiac tissues (hECTs) serve as valuable platforms for modelling both homeostasis and disease. Incorporating macrophages into hECTs significantly enhances contractile force through macrophage-derived adrenaline activating β-adrenergic signalling [171]. Similar findings were reported using an engineered cardiac microtissue containing fibroblasts and cardiomyocytes co-cultured with hESC-derived macrophages, driving sarcomeric maturation by detoxifying the cardiac microenvironment via efferocytosis [172]. Further innovations have incorporated primitive-like macrophages and endothelial cells into a microfluidic system, demonstrating that macrophages not only enhance cardiac function but also facilitate the formation of perfusable vessels [14].

Self-organising organoid models complement engineered tissues by enabling the study of developmental processes [173]. The integration of hPSC-derived macrophages pioneered in brain and intestinal organoids has revealed parallels across different organ systems. These tissue resident macrophages phenotypically resemble their foetal counterparts and perform critical functions in tissue homeostasis, modulating neural progenitor proliferation in brain organoids and glucose metabolism in intestinal organoids [174,175]. Despite the established importance of tissue resident macrophages in maintaining cardiac development and homeostasis, most cardiac organoid models to date lack an immune compartment. These include self-organising cardioids that model chamber morphogenesis and developmental processes, epicardioids that achieve epicardial differentiation through retinoic acid signalling and self-assembling heart organoids that model cardiac development and congenital heart disease through Wnt signalling modulation [176–179]. Recent work has addressed this gap by showing that integrating monocytes into cardiac organoids generates tissue resident-like macrophages that promote sarcomeric maturation and form CX43-mediated gap junctions with cardiomyocytes. These cardiac immune assembloids have been used to model arrhythmias driven by macrophage inflammation, providing insights into mechanisms underlying atrial fibrillation [15].

While of incredible valuable, these models primarily capture local immune-cardiac interactions without addressing the systemic immune responses crucial in regeneration. Organisms function as interconnected systems, where trauma triggers not only local repair but also systemic responses affecting distant tissues through circulating factors [180,181]. Body-on-a-chip systems offer a promising approach to studying these complex interactions. Multi-organ chips have been developed with hiPSC-derived heart, bone, liver and skin tissues connected by vascular flow. In these systems, circulating CD14^+^ monocytes remain in the vascular compartment under normal conditions but extravasate in response to injury signals [182,183]. These platforms enable investigation of inflammation-mediated organ-organ interactions involving circulatory systems, providing a more comprehensive understanding of regenerative processes.

Integrating immune cells into cardiac *in vitro* models enhances their ability to recapitulate the *in vivo* cardiac environment. The combination of these advanced models with single cell genomics, spatial technologies and computational modelling creates a powerful discovery pipeline for understanding how immune cells influence cardiac development and regeneration at both local and systemic levels. Future expansions incorporating microfluidic systems and multi-organ integration will likely provide deeper insights into the complex interplay between local tissue responses and systemic immune regulation during cardiac regeneration after injury.

## 7 Conclusions and future perspectives

A deep dive into the fundamental insights of immune-mediated cardiac development and regeneration holds immense potential for clinical applications. Harnessing developmental mechanisms of immune-guided cardiogenesis could inform innovative therapies for congenital heart disorders (Box 3). Modulating the immunological milieu of the damaged heart to recapitulate regenerative programmes observed in regeneration-prone model organisms and neonatal mammals offers promise for enhancing endogenous repair. Targeted immunotherapies, guided by single cell atlases and spatial maps of the cardiac immunome, may enable precise interventions to promote tissue and function restoration, while minimising fibrosis. However, the extraordinary complexity of the immune system poses challenges for therapeutic manipulation. Harnessing the regenerative potential of developmental and evolutionary mechanisms requires careful consideration of the context-dependent nature of immune cell function and the fine-tuned balance between beneficial and pathological inflammation.

**Fig. 4:**
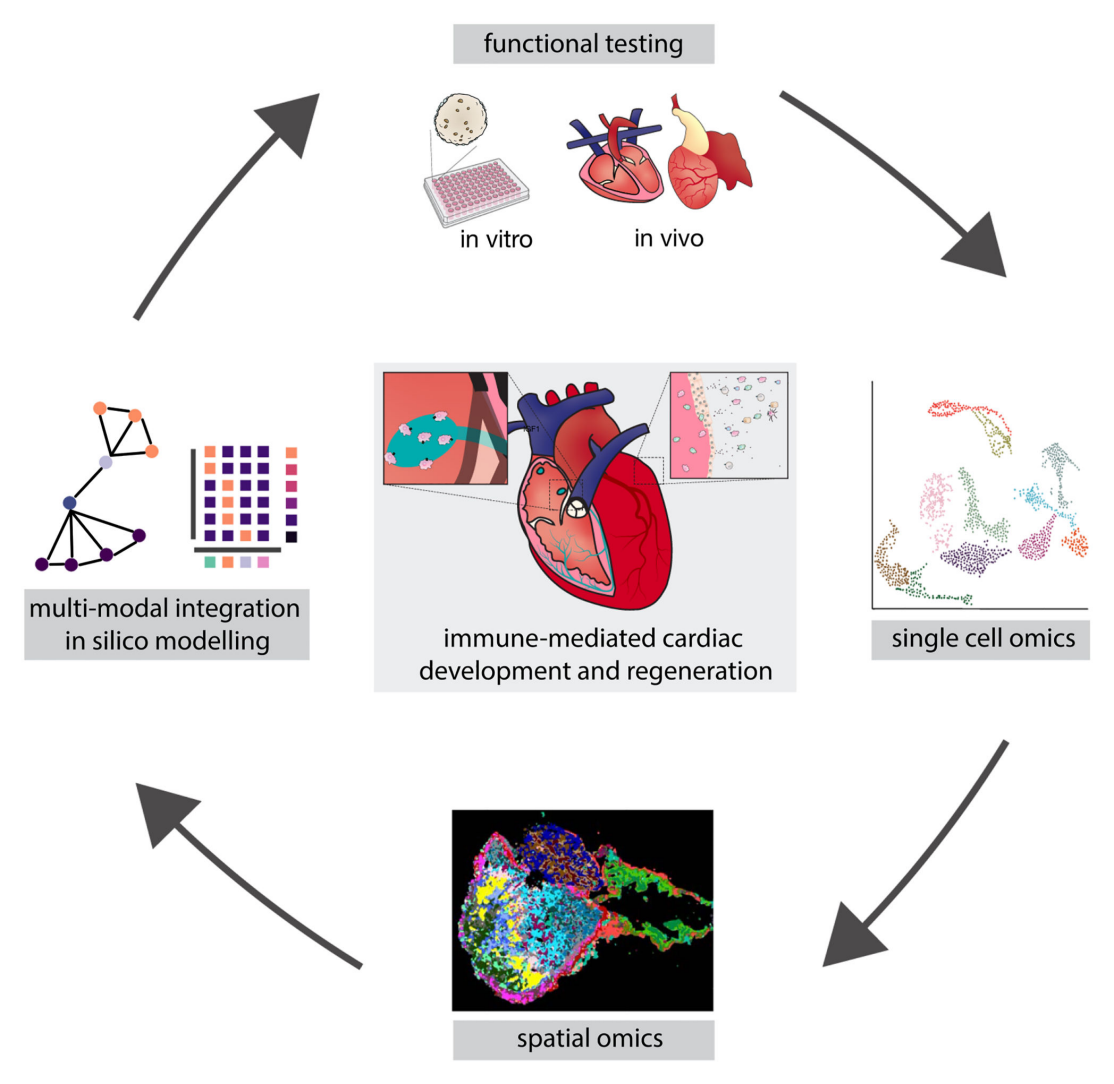
Discovering immune-mediated mechanisms in cardiac development and regeneration. Overview of a multi-step workflow, including generation and integration of multi-modal single cell datasets, providing a detailed view of diverse immune populations within the heart. The integrated single cell data is then combined with spatial information to map immune cell types and their localisation within the cardiac tissue. Building upon this, *in silico* modelling generates hypotheses about immune cell roles and interactions in regulating cardiac development and regeneration. This iterative approach allows continuous refinement and validation of these predictions through targeted *in vitro* and *in vivo* functional testing. By integrating complementary data and experiments, this pipeline aims to uncover immunemediated mechanisms orchestrating cardiac changes over development, homeostasis and regeneration.

Future research must prioritise the integration of multi-modal single cell data, spatial information and functional validation to construct a comprehensive atlas of the cardiac immunome across development, homeostasis and regeneration (Fig. 4). Comparative studies across species will provide key insights into the fundamental principles governing immune-cardiac interactions. Computational biology and machine learning will be instrumental in distilling priority therapeutic targets emerging from these complex datasets. Innovative model systems that recapitulate human cardiac immunology, such as engineered heart tissues, organoids and chimeric models will be essential for translational progress. By coupling *ex vivo* and *in vitro* approaches with *in vivo* studies in genetically tractable organisms, we will accelerate the discovery and validation of novel therapeutic strategies.

## Box 1 Haematopoietic origins of cardiac macrophages.

Cardiac macrophages demonstrate distinct subsets emerging from different haematopoietic waves. Recent studies propose a unified classification across organs, including three major cardiac populations: TLF^+^ (TIMD4, LYVE1, FOLR2), CCR2^+^ and MHC-II^high^ macrophages [16].

The development of cardiac macrophages occurs through three distinct waves of embryonic haematopoiesis: primitive haematopoiesis, erythromyeloid progenitor (EMP) wave and definitive haematopoiesis [17,18]. Primitive haematopoiesis initiates in extra-embryonic yolk sac blood islands (E7.0 in mice, week 3 in humans), generating unipotent myeloid progenitors which are restricted to the macrophage lineage [17, 19–21]. This process depends on colony stimulating factor 1 (CSF1) and its receptor (CSF1R) but occurs independently of the transcription factor c-Myb, which distinguishes it from later waves [17,22]. Once blood circulation begins (~E8.5-E9), these progenitors differentiate into primitive macrophages and seed embryonic tissues, notably establishing microglia populations in the brain and contributing to establishing cardiac resident macrophages, as shown in recent lineage tracing studies [23,24]. The second wave emerges at E8.25 from the yolk sac haemogenic endothelium, specialised vascular endothelial cells with blood-forming capacity [17,25]. This wave produces c-Myb-dependent erythromyeloid progenitors (EMPs) that can either differentiate directly into macrophages in the yolk sac or seed the foetal liver to generate monocytes. EMPs give rise to most tissue resident macrophages, including cardiac TLF^+^ populations [26,27]. Definitive haematopoiesis begins in the intra-embryonic Aorta-Gonad-Mesonephros (AGM) region (E10.5 in mice, week 5 in humans) [18,25,26,28]. Foetal haematopoietic stem and progenitor cells then migrate to the liver for transient haematopoiesis before establishing definitive bone marrow haematopoiesis postnatally [29,30]. Both transient and definitive waves generate CCR2^+^ macrophages. In adults, these CCR2^+^ populations require continuous monocyte replenishment, while TLF^+^ and MHC-II high macrophages mainly self-maintain through local proliferation [16].

## Box 2 The haemogenic endocardium: a non-classical source of immune cells.

The haemogenic potential of the endocardium remains a topic of ongoing research and debate. Early studies in mice provided initial evidence suggesting the endocardium’s ability to generate immune cells. Mouse heart explants at around E8.5 demonstrated the ability to autonomously generate myeloid and erythroid colonies [65]. Further analysis isolating CD31^+^ CD41^-^ CD45^-^ cells in the heart at E9.5 and E10.5 demonstrated expression of haematopoietic genes such as Klf1, Cbfa253, Kit, Mll1, Pbx1 and Scl/Tal1 [65]. Furthermore, colony assays also revealed that isolated endocardial cells develop myeloid and rare erythroid colonies [65]. However, more recent studies have challenged these findings, using advanced lineage-tracing techniques to reassess the endocardial specificity of previous experiments. A study incorporating more specific markers found minimal labelling of cardiac macrophages or circulating blood cells *in vivo* and no evidence of endocardial haemogenic activity in *ex vivo* cultures. Instead, the macrophages previously thought to be endocardial-derived were shown to originate from haemogenic endothelial cells in the yolk sac and aorta-gonad-mesonephros (AGM), which share Nfatc1 expression with the endocardium [24]. Similarly, lineage-tracing techniques targeting spatially restricted populations based on cell-cell contacts also found no evidence supporting the haemogenic potential of the endocardium [66]. However, this study could not trace cells in the atrioventricular and outflow cushions, where haemogenic endocardial cells were initially observed [65]. In contrast to these findings in mice, studies in zebrafish have provided support for the haemogenic potential of the endocardium. Time-lapse imaging and lineage tracing showed spi1b^+^ cells detaching from the endocardial sheet, suggesting active haematopoietic activity of the endocardium [67]. Genetic lineage tracing demonstrated that a significant portion of neutrophils observed at 24 h post-fertilisation originated from the endocardium, however little to no contribution to the macrophage lineage was reported. Furthermore, Hedgehog signalling was shown to be critical for the development of the myeloid cells of endocardial origin, contrasting the regulatory pathways involved general haematopoietic and vascular development [67]. In summary, the role of the endocardium in haematopoiesis remains complex and context-dependent, with these studies highlighting the need for novel technological approaches to further elucidate the molecular and cellular mechanisms underlying endocardial haematopoiesis and its contribution to cardiac-specific immune functions.

## Box 3 Therapeutic implications of immune-mediated cardiac development.

Understanding immune cell contributions to cardiac development offers crucial insights for both congenital and acquired heart diseases. Congenital heart defects represent the most common birth defect worldwide, affecting approximately 1 % of live births globally [184,185]. These defects arise predominantly during weeks 3–7 of human development, coinciding with critical immune cell recruitment and activation. Developmental immune mechanisms influence multiple cardiac processes, as evidenced across various pathologies. Mitral valve dystrophy (MVD), affecting 2–3 % of the global population, provides a key example. Studies using a FLNA-mutation MVD rat model revealed valve remodelling begins by postnatal day 7, preceded by inflammatory changes, with altered valve interstitial cells showing increased ICAM1 expression and enhanced monocyte adhesion [185]. Similarly, Axin2 knockout mice exhibit myxomatous valve defects with increased CCR2^+^ monocyte-derived macrophages and CD206^+^ macrophages, colocalising with immunogenic hyaluronan remodelling, a pattern also observed in human myxomatous valves [186]. Beyond these valve-specific examples, developmental immune mechanisms influence multiple cardiac processes. Macrophage populations established during development are crucial for valve formation, coronary vessel development, cardiac rhythm establishment and cardiomyocyte maturation, and therefore may contribute to various cardiac pathologies when dysregulated. This extends to maternal-foetal immune interactions. Preeclampsia, associated with increased congenital heart defect risk, involves enhanced neutrophil activation [187,188]. In murine preeclampsia models, neutrophil depletion and subsequent repopulation triggers pro-inflammatory phenotypes, particularly in placental neutrophils expressing TNFα, CXCR2, CD114 and MMP9. This neutrophil-driven inflammation activates F4/80^+^ Ly6C^high^ macrophages, enabling maternal leukocyte infiltration into the embryonic heart and creating an inflammatory microenvironment that persists postnatally [189]. Recent multiomic analyses of human embryonic and foetal hearts reveal specific immune-cardiac developmental interactions. Comparison of euploid and Trisomy 21 hearts, the latter showing 50 % prevalence of congenital heart disease, demonstrates reduced compact myocardium [6,190]. Three immune populations show significant alterations: natural killer cells, LYVE1^+^ macrophages and ATF3^+^ macrophages. Analysis reveals altered signalling between macrophages and cardiomyocytes, with increased HBEGF-ERBB4 signalling from LYVE1^+^ macrophages and decreased SPP1-Integrin αvβ3 signalling from CX3CR1^+^ macrophages to compact cardiomyocytes [6]. These insights suggest therapeutic opportunities.
